# Impact of Body Mass Index on Perioperative Outcomes in Robotic-Assisted Total Hysterectomy: A Retrospective Cohort Study

**DOI:** 10.3390/jcm15135067

**Published:** 2026-06-29

**Authors:** Zeynep Atas Elfrink, Fabinshy Thangarajah, Rainer Kimmig, Roland Csorba

**Affiliations:** Department of Gynecology and Obstetrics, University Hospital Essen, Hufelandstraße 55, 45147 Essen, Germany

**Keywords:** robotic surgery, hysterectomy, body mass index, obesity, perioperative outcomes, minimally invasive surgery, gynecology, laparoscopy

## Abstract

**Background/Objectives**: The rising global prevalence of obesity presents an increasing challenge in gynecologic surgery. Although robotic-assisted hysterectomy is widely used, comparative data on perioperative outcomes across body mass index (BMI) categories remain limited. We evaluated whether higher BMI is associated with longer operative duration and increased perioperative complications in robotic-assisted total hysterectomy performed for benign indications. **Methods**: This retrospective cohort study analyzed 179 patients who underwent robotic-assisted hysterectomy at a German academic medical center between January 2018 and December 2024. Patients were stratified by World Health Organization criteria into normal weight (BMI < 25 kg/m^2^; *n* = 51), overweight (BMI 25.0–29.9 kg/m^2^; *n* = 59), and obese (BMI ≥ 30 kg/m^2^; *n* = 69) groups. The primary outcome was operative time; secondary outcomes included estimated blood loss (EBL), Clavien–Dindo complications, hospital stay, transfusion, and readmission within six weeks. Multivariable regression adjusted for uterine weight, surgeon volume, ASA class, year of surgery, and prior abdominal operations. **Results**: Operative time increased significantly with BMI (normal: 136.3 ± 68.7 vs. obese: 174.4 ± 74.3 min; *p* = 0.009). On multivariable analysis, BMI remained an independent predictor of operative time (*β* = 2.49 min per kg/m^2^, 95% CI 1.01–3.96, *p* = 0.001) and EBL (*β* = 15.0 mL per kg/m^2^, 95% CI 1.5–28.5, *p* = 0.029). Postoperative hemoglobin and transfusion rates did not differ between groups. No significant differences were detected in major complication rates (Clavien–Dindo ≥ III: 4/51 [7.8%], 1/59 [1.7%], 7/69 [10.1%]; *p* = 0.15), hospital stay, or readmission. High-volume surgeon status (≥30 cases) was independently associated with reduced major complications (OR = 0.16, 95% CI 0.03–0.75, *p* = 0.020). **Conclusions**: Robotic-assisted hysterectomy appears clinically feasible across all BMI categories without a detectable increase in major morbidity, although obesity was associated with moderately longer operative times and higher calculated EBL. The study was not powered to detect differences in rare events. Surgeon experience was independently associated with lower complication rates and may help offset the additional technical demands of obesity.

## 1. Introduction

Obesity is a major global health challenge with far-reaching surgical implications. The worldwide prevalence has more than tripled since 1975, with an estimated one billion people currently living with obesity [1,2]. According to World Health Organization criteria, adults are classified as normal weight (BMI 18.5–24.9 kg/m^2^), overweight (BMI 25.0–29.9 kg/m^2^), or obese (BMI ≥ 30 kg/m^2^) [3]. The prevalence of obesity continues to rise across all age groups and is increasingly seen among patients presenting for gynecologic surgery.

Hysterectomy is one of the most frequently performed gynecologic procedures, with more than 400,000 inpatient procedures recorded annually in the United States alone [4]. Over the past two decades, the surgical approach has shifted substantially from open abdominal surgery toward minimally invasive techniques. Robotic-assisted surgery has emerged as an important approach, offering enhanced three-dimensional visualization, tremor filtration, improved instrument articulation, and ergonomic advantages for the operating surgeon [5,6]. A recent Cochrane review found that in benign and malignant gynecologic disease, robotic-assisted surgery achieved outcomes broadly comparable to conventional laparoscopy, with lower conversion rates to laparotomy in some settings [7].

Obesity poses specific challenges in robotic gynecologic surgery. Steep Trendelenburg positioning, required for adequate pelvic exposure, can compromise pulmonary mechanics in patients with increased BMI; trocar placement through a thicker abdominal wall and the additional torque applied by robotic arms can complicate port positioning and increase the risk of port-site complications [8]. In addition, obesity is associated with chronic low-grade inflammation, altered tissue perfusion, and a higher prevalence of medical comorbidities such as diabetes, hypertension, and obstructive sleep apnea, all of which contribute to perioperative risk [8,9]. Despite these concerns, several studies have suggested that the technical advantages of robotic-assisted surgery—particularly improved visualization and instrument precision in the deep pelvis—may be particularly beneficial in obese patients, where conventional laparoscopy can be technically demanding [9,10].

Published evidence comparing perioperative outcomes across BMI categories specifically in robotic-assisted hysterectomy remains limited. Haveman and colleagues analyzed 356 robotic-assisted total laparoscopic hysterectomies stratified by WHO obesity classes and found that operative time was significantly longer only in patients with BMI ≥ 40 kg/m^2^, while complication and conversion rates were similar across all classes [11]. A meta-analysis by Cusimano and colleagues including more than 6000 obese patients with endometrial cancer demonstrated lower conversion rates and comparable complication rates with robotic-assisted compared with conventional laparoscopic hysterectomy [12]. Iavazzo and colleagues, in a three-year experience of robotic gynecologic surgery in 93 obese women, reported no conversions to laparotomy and a low complication rate [13]. Despite these encouraging findings, evidence from European academic centers covering the full BMI spectrum in benign disease remains scarce, and the independent contribution of BMI after adjustment for confounders such as uterine weight and surgeon volume has not been consistently characterized [11,12,14].

The Department of Gynecology and Obstetrics at University Hospital Essen has performed robotic-assisted gynecologic surgery for more than 15 years and is recognized as a high-volume European center. This study aimed to evaluate whether higher BMI is associated with longer operative duration and increased perioperative complication rates in robotic-assisted total hysterectomy for benign indications, with adjustment for uterine weight, surgeon volume, and other relevant covariates.

## 2. Materials and Methods

### 2.1. Study Design and Setting

This was a single-center retrospective cohort study conducted at the Department of Gynecology and Obstetrics, University Hospital Essen, Germany. The study was reported in accordance with the STROBE guideline for cohort studies and was approved by the Institutional Review Board of the Medical Faculty of the University of Duisburg-Essen (protocol code 25-12723-BO, approved 1 October 2025).

### 2.2. Patient Selection

Using institutional ICD and procedural codes (OPS 5-683), we identified all patients who underwent robotic-assisted hysterectomy between 1 January 2018 and 31 December 2024 (*n* = 1479). Inclusion criteria were age 18–90 years, robotic-assisted total laparoscopic hysterectomy with bilateral salpingectomy or salpingo-oophorectomy, a benign indication (uterus myomatosus, abnormal uterine bleeding, endometriosis/adenomyosis, cervical intraepithelial neoplasia, complex benign ovarian cysts, or endometrial hyperplasia), and complete documentation of BMI and pre-, intra-, and postoperative parameters. Exclusion criteria were histologically confirmed gynecologic malignancy, supracervical hysterectomy, concomitant urogynecologic procedures (vaginal repair, urethral suspension, sacrocolpopexy), emergency surgery, combined procedures with other surgical disciplines, and incomplete records for primary endpoints. To account for the surgical learning curve, the first 20 operations of each of the six surgeons were excluded; this threshold reflects the early learning curve described in the literature for robotic hysterectomy, which typically reaches an initial plateau after 15–25 cases [15]. After all exclusions, 179 patients were eligible.

### 2.3. Surgical Procedure

All patients underwent total hysterectomy with bilateral salpingectomy or salpingo-oophorectomy using the da Vinci three-armed or four-armed robotic platform (Intuitive Surgical Inc., Sunnyvale, CA, USA) with standardized techniques. Surgeries were performed by six surgeons with varying case volumes ranging from 12 to 60 procedures during the study period, after the exclusion of the first 20 operations of each of the six surgeons.

### 2.4. Data Collection and Outcomes

Data were extracted from the institutional electronic medical record (MEDICO^®^, CompuGroup Medical SE & Co. KGaA, Koblenz, Germany), including operative reports, pathology reports, anesthesia records, laboratory results, and discharge summaries. The following data were collected:

Preoperative: age, height, weight, BMI, indication for hysterectomy, ASA classification, smoking status, number of prior abdominal operations, preoperative hemoglobin and hematocrit, and preoperative leukocyte count.

Intraoperative: type of procedure, operative time, intraoperative complications, conversion to laparotomy, and abdominal versus vaginal retrieval of the uterine specimen. Operative time was defined as the total anesthesia duration from induction to emergence, as recorded in the structured anesthesia record (Anästhesieprotokoll). This definition was chosen for three reasons. First, anesthesia start and end timestamps are recorded consistently across the seven-year study period. Second, in robotic surgery, the additional time required for steep Trendelenburg positioning, port placement, and robot docking is surgically meaningful and should not be excluded from a measure of operative burden. Third, total anesthesia duration corresponds to the patient’s physiological exposure to pneumoperitoneum and Trendelenburg positioning, which is the clinically relevant exposure when considering perioperative risk in higher-BMI patients. We acknowledge that this definition differs from the skin-to-skin convention used in much of the published literature and discuss the implications in Section 4.9.

Postoperative: postoperative hemoglobin and hematocrit, calculated EBL, postoperative C-reactive protein and leukocyte count, transfusion requirement, antibiotic use, hospital length of stay, intensive care admission, postoperative complications graded according to the Clavien–Dindo classification [16], reoperation within 42 days, and readmission within 6 weeks.

Patients were stratified according to WHO criteria into normal weight (BMI < 25 kg/m^2^), overweight (BMI 25.0–29.9 kg/m^2^), and obese (BMI ≥ 30 kg/m^2^) groups [3]. For analysis, complications were grouped into minor (Clavien–Dindo grade I–II) and major (grade ≥ III). The primary outcome was operative time; secondary outcomes were EBL, transfusion requirement, hospital length of stay, postoperative inflammation markers, major complications, and readmission.

### 2.5. Estimated Blood Loss Calculation

EBL was calculated using a hematocrit-based formula rather than visual estimation, which has been shown to systematically underestimate intraoperative blood loss in minimally invasive surgery [17,18]. We used the dilution-corrected formula:

EBL (mL) = EBV × (Hct_pre − Hct_post)/Hct_mean

where the estimated blood volume (EBV) was calculated as 65 mL/kg × body weight and Hct_mean is the average of the preoperative and postoperative hematocrit values. Preoperative laboratory values within two weeks before surgery and postoperative values within two days after surgery were used.

A methodological consideration of this formula is that EBV scales linearly with body weight, which may inflate the calculated EBL in obese patients for equivalent proportional hematocrit changes; standard EBV estimates have been shown to overestimate true blood volume in patients with obesity [19]. We address this in the limitations and discussion, and we report postoperative hemoglobin alongside calculated EBL to allow readers to assess this potential artifact.

### 2.6. Statistical Analysis

Continuous variables were tested for normality using the Shapiro–Wilk test and are presented as mean ± standard deviation (SD) or median (interquartile range, IQR) as appropriate. Categorical variables are presented as absolute frequencies and percentages.

Group comparisons used one-way analysis of variance (ANOVA) for normally distributed continuous variables and the Kruskal–Wallis test for non-normally distributed variables. Post hoc pairwise comparisons used the Dunn test with Bonferroni correction. Categorical variables were compared using the χ^2^ test, or Fisher’s exact test when expected cell frequencies were below 5. Spearman’s rank correlation coefficient (ρ) was calculated to assess associations between continuous variables, including BMI and primary outcomes.

Multivariable analyses included two linear regression models (for operative time and EBL) and one logistic regression model (for major complications, Clavien–Dindo ≥ III). All models included BMI as a continuous variable, uterine weight, surgeon volume (high-volume defined as ≥30 cases during the study period versus lower-volume), ASA class, year of surgery, and a history of ≥2 prior abdominal operations. The ≥30 case threshold for high-volume surgeon designation was chosen to reflect the operative volume associated with reduced complication rates in the published gynecologic surgery literature [20] while remaining conservative given the lower published proficiency thresholds (up to 75 cases) described for full robotic hysterectomy mastery [21]. We acknowledge that this operational cutoff is lower than full proficiency benchmarks and is specific to the case volumes attainable within our department over the study period; its rationale and limitations are discussed further in Section 4.6.

Multivariable models were performed on patients with complete data; uterine weight was available for 153 out of 179 patients (85.5%). The proportion of missing uterine weight differed across BMI groups (5.9% in normal weight versus 18.8% in obese patients), and this is acknowledged as a potential source of bias. Detailed information on adhesion severity, endometriosis burden, and adenomyosis was not systematically recorded and could not be included in the models. Effect sizes for continuous outcomes were quantified using Cohen’s d and η^2^, with d = 0.2 considered small, 0.5 medium, and 0.8 large [22]. Statistical significance was set at a two-sided *p* < 0.05. No a priori sample size calculation was performed because of the descriptive nature of the analysis; the power implications are discussed in Section 4.9. All analyses were performed using Python 3.13 (Python Software Foundation, Wilmington, DE, USA) with the SciPy, statsmodels, and scikit-posthocs packages.

## 3. Results

### 3.1. Patient Characteristics

Of the 1479 robotic-assisted gynecologic procedures performed during the study period, 179 patients met the inclusion criteria. The cohort comprised 51 normal weight (28.5%), 59 overweight (33.0%), and 69 obese (38.5%) patients, with a mean BMI of 29.2 ± 7.3 kg/m^2^ (range 19–59). Demographic and preoperative characteristics are summarized in Table 1. Groups were comparable in age (49.1–50.2 years, *p* = 0.72), ASA classification (*p* = 0.34), smoking status (*p* = 0.75), number of prior abdominal operations (*p* = 0.642), and uterine weight (*p* = 0.618). Although there was a trend toward higher ASA III prevalence in the obese group (27.5% vs. 11.8% in normal weight), this did not reach statistical significance.

The most common indication was uterus myomatosus (81.6%, *n* = 146), followed by cervical intraepithelial neoplasia (7.8%, *n* = 14), abnormal uterine bleeding (3.4%, *n* = 6), endometrial hyperplasia (3.4%, *n* = 6), and other benign conditions including ovarian cyst, endometriosis, and adenomyosis (3.9%, *n* = 7). The annual number of procedures increased over the study period, with a notable decline in 2020 attributable to the COVID-19 pandemic.

### 3.2. Perioperative Outcomes

**Figure 1:**
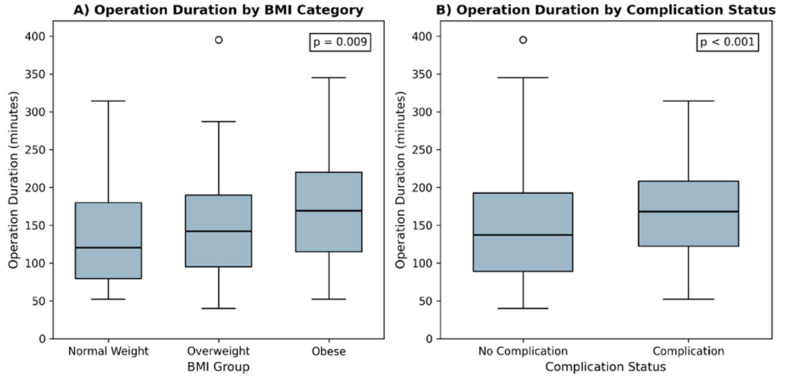
(**A**) Operative time by BMI group. (**B**) Operative time by occurrence of any postoperative complication.

Operative time increased significantly with increasing BMI (Spearman ρ = 0.280, *p* < 0.001). Mean operative time was 136.3 ± 68.7 min in normal weight, 146.7 ± 67.3 min in overweight, and 174.4 ± 74.3 min in obese patients (Table 2; Figure 1A). Post hoc analysis with Bonferroni correction showed a significant difference between normal weight and obese groups (mean difference 38.1 min, 95% CI 11.7–64.5, *p* = 0.009), while differences between adjacent groups did not reach significance after correction (normal vs. overweight *p* = 1.00; overweight vs. obese *p* = 0.12). The effect size of the overall group difference was small to moderate (η^2^ = 0.052; Cohen’s d for normal vs. obese = 0.53). Patients experiencing any complication had longer operations (median 160 vs. 120 min, *p* < 0.001; Figure 1B). The direction of this association cannot be determined from the present data: a longer operative time may reflect the additional intraoperative management required when a complication arises (e.g., hemostasis, conversion preparation), or alternatively, prolonged exposure to pneumoperitoneum, steep Trendelenburg, and tissue manipulation may itself increase the complication risk; these mechanisms are not mutually exclusive.

**Figure 2:**
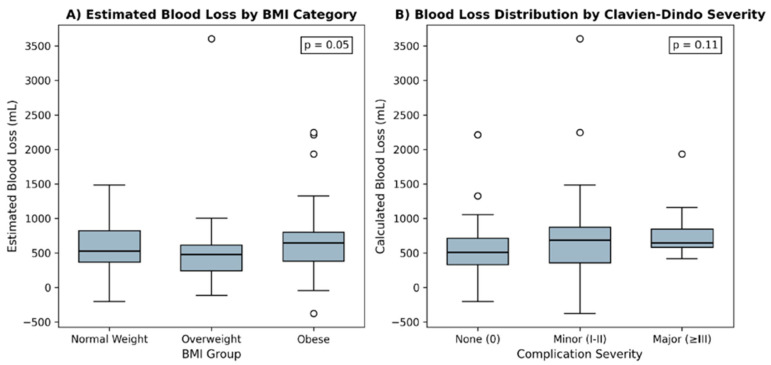
(**A**) Estimated blood loss by BMI category. (**B**) Blood loss distribution by Clavien-Dindo complication severity.

Calculated EBL was significantly higher in obese versus normal weight patients (824.3 ± 652.3 vs. 600.8 ± 346.9 mL; mean difference 223.5 mL, 95% CI 34.3–426.3, *p* = 0.029; Table 2; Figure 2A). The absolute EBL values are higher than those typically reported for routine robotic hysterectomy, reflecting both the objective hematocrit-based calculation method and the complex case mix inherent to a tertiary referral center. In a subgroup analysis stratified by prior abdominal surgery, EBL differed significantly across BMI groups among patients with prior abdominal surgery (*p* = 0.029) but not among those without (*p* = 0.088). Notably, the postoperative hemoglobin values did not differ between BMI groups (11.4 vs. 11.3 vs. 11.4 g/dL; *p* = 0.87), and blood transfusion was required in 9 patients (5.0%) with no difference among groups (5.9% normal weight, 5.1% overweight, 4.3% obese; *p* = 0.93). This dissociation between calculated EBL and the clinical markers of blood loss is discussed in Section 4.3.

Hospital stay was similar across groups (median 4.0 days in normal weight and overweight vs. 3.0 days in obese; *p* = 0.24). Postoperative C-reactive protein was significantly higher in obese patients (4.94 ± 4.99 vs. 2.67 ± 3.13 mg/dL; *p* = 0.004), whereas the postoperative leukocyte increase did not differ significantly between groups (*p* = 0.96).

### 3.3. Complications

Postoperative complications occurred in 51 patients (28.5%), with no significant difference among BMI groups (*p* = 0.91; Table 3). Major complications (Clavien–Dindo ≥ III) occurred in 12 patients (6.7%): 7.8% in normal weight, 1.7% in overweight, and 10.1% in obese patients (*p* = 0.15). Of note, this distribution is non-monotonic, with the overweight group showing the lowest major complication rate. Given the small absolute numbers of major complications in each group (*n* = 4, 1, and 7), this pattern is most plausibly explained by sampling variation rather than a true protective effect of overweight status; nonetheless, it limits the strength of any conclusion about a monotonic BMI–complication relationship.

Clavien–Dindo grade III complications (*n* = 10, 5.6%) included vaginal cuff hematoma requiring operative evacuation (*n* = 4), delayed vaginal bleeding with reintervention (*n* = 2), abdominal hematoma with wound revision (*n* = 2), and postoperatively identified bladder injury managed with cystoscopy under local (grade IIIa, *n* = 1) or general anesthesia (grade IIIb, *n* = 1). Two patients (1.1%) required intensive care admission (grade IV); both occurred in the normal weight group. No perioperative deaths occurred. Reoperation under general anesthesia was required in 9 patients (5.0%), most commonly for vaginal cuff hematoma; the difference between groups (5.9% normal weight, 0% overweight, 8.7% obese) did not reach statistical significance (*p* = 0.08). Readmission within six weeks occurred in 13 patients (7.3%), with no significant difference between BMI groups (*p* = 0.33).

Conversion to laparotomy was required in 4 out of 179 patients (2.2%). Three conversions occurred in the overweight group (driven by large myoma size precluding safe robotic preparation in two cases and intraoperative complications in one) and one in the normal weight group; no conversion occurred in the obese group.

**Figure 3:**
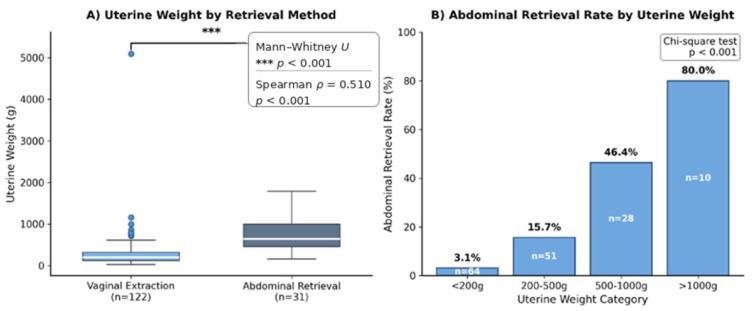
(**A**) Uterine weight by retrieval method. (**B**) Abdominal retrieval rate by uterine weight category.

Abdominal specimen retrieval via minilaparotomy was required in 31 out of 153 patients with documented uterine weight (20.3%) and correlated strongly with uterine weight (Spearman ρ = 0.510, *p* < 0.001; Figure 3). The abdominal retrieval rate rose from 3.1% in uteri < 200 g to 80.0% in uteri > 1000 g (χ^2^ *p* < 0.001). Although uterine weight itself did not differ significantly between BMI groups (*p* = 0.618), the abdominal retrieval rate was higher in obese patients (29.0%) than in normal weight (21.6%) or overweight (10.2%) patients (*p* = 0.03), suggesting that anatomical factors beyond uterine weight—such as reduced vaginal accessibility from perineal and paravaginal adiposity—contribute to the choice of retrieval route in obese patients.

### 3.4. Multivariable Analysis

On multivariable linear regression (*n* = 153 with complete data), BMI remained an independent predictor of operative time (β = 2.49 min per kg/m^2^, 95% CI 1.01–3.96, *p* = 0.001), as did uterine weight (β = 0.03 min/g, *p* = 0.006) and high-volume surgeon status (β = −28.6 min, *p* = 0.019). The model explained approximately 18% of the variance in operative time (adjusted R^2^ = 0.181), meaning that more than 80% of the between-patient variability in operative time remained unexplained by BMI, uterine weight, surgeon volume, ASA class, year of surgery, and prior abdominal operations combined. The BMI coefficient should therefore be interpreted as a real but modest contributor among many unmeasured determinants—likely including adhesion severity, endometriosis burden, anatomical variation, and team-specific workflow factors that we could not capture retrospectively—rather than as a dominant driver of operative duration. For calculated EBL (*n* = 134), BMI (β = 15.0 mL per kg/m^2^, *p* = 0.029) and uterine weight (β = 0.22 mL/g, *p* = 0.016) were independent predictors (adjusted R^2^ = 0.088); the model thus accounted for less than 10% of the variance in EBL, and the same interpretive caution applies as for operative time. On logistic regression for major complications, BMI was not a significant predictor (OR = 0.98, *p* = 0.679); the only significant predictor was high-volume surgeon status (OR = 0.16, 95% CI 0.03–0.75, *p* = 0.020).

### 3.5. Surgeon Volume

The six surgeons performed between 12 and 60 procedures each during the study period. High-volume surgeons (≥30 cases, *n* = 126 procedures) demonstrated significantly lower overall major complication rates than lower-volume surgeons (3.2% vs. 15.1%, *p* = 0.007). Within the obese subgroup, major complications occurred in 1 out of 37 obese patients (2.7%) operated by high-volume surgeons compared with 6 out of 32 (18.8%) operated by lower-volume surgeons (*p* = 0.044). The wide confidence interval around this estimate (Section 4.6) reflects the small number of events.

## 4. Discussion

### 4.1. Principal Findings

The principal finding of this study is that robotic-assisted total hysterectomy was clinically feasible across all BMI categories in our cohort, with no statistically significant difference in major complication rates between groups. BMI was independently associated with longer operative times and higher calculated EBL on multivariable analysis, but these differences did not translate into significantly higher rates of transfusion, prolonged hospital stay, or major morbidity. Surgeon volume was the only significant independent predictor of major complications, suggesting that operator experience may help mitigate the additional technical demands of robotic surgery in obese patients. The absence of a statistically significant complication difference between BMI groups should not be interpreted as equivalence: with only 12 major complications across the cohort, the study was underpowered to detect clinically meaningful differences in rare events, and the observed numerical pattern (7.8% vs. 1.7% vs. 10.1%) was non-monotonic. Because operative time was measured as total anesthesia duration, the BMI–time association should be interpreted as reflecting total perioperative burden rather than surgical difficulty alone (Section 4.9).

### 4.2. Comparison with Comparable Cohorts

Our findings are most directly comparable to those of Haveman and colleagues, who analyzed 356 robotic-assisted total laparoscopic hysterectomies stratified by WHO obesity classes [11]. They reported that operative time was significantly longer only in patients with BMI ≥ 40 kg/m^2^, while complication and conversion rates did not differ between BMI groups. Our results are consistent with theirs in terms of the safety profile across BMI categories, although we found that the operative-time effect of BMI was already detectable at the group level when comparing normal weight to obese patients overall. The difference may reflect the heterogeneity of our obese subgroup, which included 33 patients with a BMI ≥ 35 kg/m^2^ and 10 with BMI ≥ 40 kg/m^2^, or differences in case mix and surgeon experience between centers.

Kissane and colleagues, in contrast, found no significant difference in operative time across BMI categories in robotic-assisted sacrocolpopexy (202, 206, and 216 min for normal weight, overweight, and obese; *p* = 0.53) [9]. The discrepancy between these findings and ours may reflect procedural differences: sacrocolpopexy is more standardized in its operative steps, whereas total hysterectomy includes variable steps (specimen morcellation, retrieval method, hemostasis at the vaginal cuff) where additional technical difficulty in obese patients can accumulate. Bradley and colleagues previously demonstrated longer operative times in obese versus normal weight women undergoing open abdominal sacrocolpopexy (189 vs. 169 min, *p* = 0.02) [23]; that the same effect may not appear in robotic sacrocolpopexy supports the hypothesis that the robotic platform partially compensates for obesity-related technical challenges. Iavazzo and colleagues, reporting a three-year experience in 93 obese women undergoing robotic gynecologic surgery, observed a mean operative time of 150 min and a 0% conversion rate [13], consistent with the broader trend that obesity is not a barrier to robotic surgery in experienced hands.

The recent meta-analysis by Cusimano and colleagues of laparoscopic versus robotic hysterectomy in obese patients with endometrial cancer reported lower conversion rates and comparable major complication rates for the robotic approach [12]. The mega-meta-analysis by Lenfant and colleagues, including more than 1.1 million patients, similarly found that robotic-assisted hysterectomy was associated with shorter hospital stay, lower blood loss, and fewer complications compared with open surgery, with equivalent outcomes to conventional laparoscopy [24]. Our cohort, although smaller, fits within the contours of this larger literature.

### 4.3. Calculated Blood Loss and the Limits of the Formula

The significantly higher calculated EBL in obese patients warrants careful interpretation. We used a hematocrit-based formula because visual estimation systematically underestimates intraoperative blood loss, particularly in minimally invasive surgery where blood is diluted with irrigation fluid and distributed across the operative field [17]. However, the formula uses an EBV of 65 mL/kg × body weight, which scales linearly with weight and therefore yields a higher absolute calculated EBL in heavier patients for the same proportional hematocrit change. Lemmens and colleagues have shown that standard EBV estimates overestimate true blood volume in obese patients, and several alternative formulas adjust EBV using ideal body weight [19]. Recalculating EBL using an obesity-corrected EBV would likely attenuate the BMI–EBL association observed in this study.

Several clinical observations support the interpretation that the calculated EBL difference is at least partly a formula artifact. First, postoperative hemoglobin did not differ between groups (11.4, 11.3, and 11.4 g/dL; *p* = 0.87). Second, transfusion rates were almost identical (5.9%, 5.1%, 4.3%; *p* = 0.93). Third, hospital length of stay was, if anything, shorter in the obese group. If obese patients were experiencing clinically meaningful blood loss in excess of normal weight patients, we would expect a downstream signal in at least one of these clinical markers. The fact that none is present suggests that the formula-driven increase in calculated EBL does not reflect a clinically relevant difference in true blood loss. We acknowledge this as a limitation of the analytic approach rather than as a feature of the surgical technique.

### 4.4. Inflammation and Postoperative Course

Postoperative C-reactive protein was significantly higher in obese patients (4.94 vs. 2.67 mg/dL), while the postoperative leukocyte increase did not differ. This dissociation suggests that the elevated CRP response in obese patients largely reflects chronic adipose-tissue inflammation rather than a more pronounced acute surgical stress response. The clinical implication is that postoperative CRP in obese patients should be interpreted in this context, and isolated CRP elevation in the absence of leukocytosis, fever, or other clinical signs should not be over-interpreted as a marker of acute surgical morbidity.

### 4.5. Uterine Weight, Specimen Retrieval, and Surgical Planning

Uterine weight was independently associated with operative time (β = 0.03 min/g) and EBL (β = 0.22 mL/g) on multivariable analysis. These findings are consistent with those of Higuchi and colleagues, who in a cohort of 724 patients undergoing robotic hysterectomy for benign indications reported that operative time and EBL increased significantly with uterine weight, while complication rates and length of stay were comparable across weight categories [25]. Together, these results support the routine integration of preoperative uterine size estimation into surgical planning and patient counselling.

The strong correlation between uterine weight and abdominal specimen retrieval (ρ = 0.510, *p* < 0.001), with retrieval rates rising from 3.1% for uteri < 200 g to 80.0% for uteri > 1000 g, mirrors published findings on uterine-extraction scoring [26]. Importantly, uterine weight itself did not differ between BMI groups in our cohort, yet abdominal retrieval was more frequent in obese patients (29.0% vs. 10.2% in overweight; *p* = 0.03). This pattern suggests that BMI-related anatomical factors (reduced vaginal accessibility, limited mobilization in steep Trendelenburg, more difficult manual morcellation) contribute to the choice of retrieval route independent of uterine weight. The increased rate of abdominal retrieval in obese patients did not translate into more complications or longer hospital stay, supporting the safety of pragmatic retrieval-route choices in this population.

### 4.6. Surgeon Experience and the Volume Effect

Surgeon volume was the only independent predictor of major complications in our multivariable model (OR 0.16, 95% CI 0.03–0.75, *p* = 0.020). High-volume surgeons (≥30 cases) had lower overall major complication rates (3.2% vs. 15.1%, *p* = 0.007), and this effect was particularly evident in the obese subgroup (2.7% vs. 18.8%, *p* = 0.044). The wide confidence interval reflects the small absolute number of events and the modest size of the lower-volume obese subgroup (*n* = 32). With this caveat, the magnitude of the association is consistent with the broader literature: Mowat and colleagues, in a systematic review, found that surgeons performing fewer than one minimally invasive hysterectomy per month had significantly higher rates of adverse outcomes [20], and Carbonnel and colleagues, in a decade-long single-center analysis, defined a proficiency threshold of approximately 75 cases for robotic hysterectomy, with surgeons above this threshold achieving significantly shorter operative times (157.3 vs. 178.6 min, *p* = 0.005) despite operating on more complex cases [21].

Our chosen threshold of ≥30 cases is more conservative than the 75-case proficiency benchmark but reflects the volumes feasible within a single department over the study period. It also yields subgroups of analyzable size and corresponds to the operative volume above which reduced complication rates have been reported in the broader gynecologic surgery literature [20]; applying a 75-case cutoff would have left too few surgeons above the threshold for meaningful comparison in our cohort. Because the cutoff is center- and period-specific, it should be interpreted as a cohort-specific operational definition rather than a generalizable proficiency threshold, and the optimal volume cutoff for robotic hysterectomy in obese patients remains to be defined prospectively. We interpret the surgeon-volume effect as an association rather than a causal mechanism: high-volume surgeons in our cohort may also differ from lower-volume surgeons in case selection, team familiarity, and experience with the specific platform iteration used. Although our findings are consistent with the hypothesis that experience offsets the technical demands of operating on obese patients, prospective studies would be needed to test this causal claim directly.

### 4.7. Cost Considerations

Although we did not perform a formal cost analysis, the absence of differences in transfusion, hospital stay, or readmission across BMI groups has implications for the cost discussion around robotic surgery. Robotic platforms have been criticized for high acquisition and maintenance costs [27], and economic comparisons against laparoscopy depend strongly on which fixed and variable costs are included. Baracy and colleagues found that when initial platform costs were excluded, robotic hysterectomy showed a trend toward lower surgical and overall hospital costs than conventional laparoscopy [28]. Klebanoff and colleagues, in a cohort of 600 patients stratified by BMI quintile, observed a trend toward lower costs with the robotic approach in morbidly obese patients, attributable to shorter operative time, lower EBL, and shorter length of stay [29]. Given that surgical complications are associated with 119% higher costs compared with uncomplicated cases [30], a surgical approach that reduces complications in higher-risk groups—such as the obese—could be cost-effective even at higher per-case equipment costs, particularly at high-volume centers benefiting from scale.

### 4.8. Strengths

This study has several strengths. We analyzed a consecutive cohort over a seven-year period at a high-volume European academic robotic surgery center, including a substantial proportion of obese patients (38.5%) and a meaningful representation of higher-class obesity (BMI ≥ 35: *n* = 33; BMI ≥ 40: *n* = 10). The exclusion of malignant indications, supracervical procedures, and concomitant urogynecologic operations reduced confounding from variable surgical complexity. We used standardized techniques across surgeons, objective hematocrit-based EBL calculation, and the Clavien–Dindo classification with an independent review of complications. We performed multivariable analyses adjusting for uterine weight, surgeon volume, ASA class, year of surgery, and prior surgery, allowing us to estimate the independent contribution of BMI.

### 4.9. Limitations

Several limitations should be considered when interpreting our findings. First, the sample size of 179 patients is modest, particularly for detecting differences in rare events. The observed difference in major complications (7.8% in normal weight vs. 10.1% in obese) was not statistically significant (*p* = 0.15), but this comparison was underpowered. Based on the event rates observed, an adequately powered study to detect a clinically meaningful difference of this magnitude with 80% power would require a substantially larger cohort, and our findings should therefore be interpreted as a hypothesis-generating analysis with respect to major complications. A specific caution applies to the multivariable logistic regression for major complications: it included six covariates against only 12 events, an events-per-variable ratio of approximately two that is well below the conventional minimum of ten. This raises the possibility of overfitting and of unstable, potentially exaggerated odds-ratio estimates, reflected in the wide confidence interval around the surgeon-volume effect (OR 0.16, 95% CI 0.03–0.75). The logistic model should therefore be regarded as exploratory and hypothesis-generating, and the associations it identifies require confirmation in larger cohorts with adequate event counts. Second, the calculated EBL formula incorporates body weight, which may inflate the calculated values in obese patients for equivalent proportional hematocrit changes [19]; the absence of differences in postoperative hemoglobin and transfusion supports the interpretation that this is partly an analytic artifact. Third, we lacked systematic data on adhesion severity, endometriosis burden, and adenomyosis, all of which can influence operative time and bleeding independently of BMI. These factors were not part of the structured operative documentation and could not be retrieved retrospectively; their absence represents residual confounding by surgical complexity and is consistent with the large proportion of variance left unexplained by our models (adjusted R^2^ = 0.18 for operative time and 0.09 for estimated blood loss). Fourth, uterine weight was missing in 14.5% of patients, disproportionately in the obese group (18.8% vs. 5.9% in normal weight); although we performed complete-case analysis, this may have biased the multivariable estimates. Because missingness was differential by BMI group, the complete-case estimates may be biased in either direction; we did not perform multiple imputation given the modest sample size and the absence of strong auxiliary variables predictive of missingness, and we therefore present the complete-case results transparently as a limitation. Fifth, the retrospective single-center design and tertiary referral setting limit generalizability and may introduce selection bias (arising from referral patterns and the applied inclusion and exclusion criteria) and information bias (arising from reliance on routinely recorded clinical documentation rather than prospectively standardized data capture). Sixth, we did not capture patient-reported outcomes such as pain scores, quality of life, or return to normal activity, which are important dimensions of surgical success. These were not part of the routine clinical documentation during the study period and could not be reconstructed retrospectively; we regard their prospective collection as a priority for future work (Section 4.10). An additional consideration relates to our definition of operative time as total anesthesia duration rather than skin-to-skin time. Although this choice was driven by the reliability of structured anesthesia timestamps over a seven-year retrospective period and reflects the patient’s true exposure to steep Trendelenburg positioning and pneumoperitoneum, it means that the observed BMI–operative time association may partly reflect non-surgical components of the procedure. Higher BMI is known to prolong induction (more difficult airway management, longer venous access), positioning (additional padding, more cautious transfer into steep Trendelenburg, longer time to achieve safe trocar geometry through a thicker abdominal wall), and emergence (slower extubation and recovery to a transfer-ready state). In addition, total anesthesia duration is shaped by perioperative workflow and by the experience of the attending anesthesiologist, none of which we could account for retrospectively. The β = 2.49 min per kg/m^2^ estimate from our multivariable model should therefore be interpreted as the BMI association with total operative burden, not as a pure measure of surgical complexity. Studies using skin-to-skin or console time as the outcome may report attenuated BMI effects relative to ours, and direct quantitative comparison with such studies should account for this definitional difference. A future analysis stratifying operative time into anesthesia setup, surgical console, and emergence components would help isolate where the BMI effect predominantly accrues.

### 4.10. Future Directions

Several directions for future research follow from this analysis. Adequately powered multicenter studies with standardized data collection—including adhesion scoring, endometriosis staging, and patient-reported outcomes—would substantially strengthen the evidence base for robotic surgery in obese patients. Prospective studies should incorporate standardized scoring of surgical complexity—for example, validated intraoperative adhesion scores and revised ASRM endometriosis staging—alongside uterine size, so that the independent contribution of BMI can be estimated after adjustment for these determinants. Comparative effectiveness studies including formal cost analyses and stratified outcomes by surgeon volume would help define when the robotic approach offers the greatest marginal benefit. Development and validation of preoperative scoring systems incorporating BMI, uterine weight, vaginal accessibility, and prior surgery could improve surgical planning and patient counselling. Finally, sensitivity analyses using obesity-corrected EBV formulas should become routine in studies of perioperative bleeding in this population to avoid attributing analytic artifacts to clinical phenomena.

## 5. Conclusions

In this single-center retrospective cohort of 179 patients undergoing robotic-assisted total hysterectomy for benign indications, BMI was independently associated with longer operative times and higher calculated EBL, but these differences did not translate into significantly higher rates of major complications, transfusion, or prolonged hospitalization. Conversion to laparotomy was rare (2.2%) and occurred only in normal weight and overweight patients. Surgeon volume was the only independent predictor of major complications, with high-volume surgeons achieving substantially lower complication rates, particularly in obese patients. These findings, considered alongside the published comparable cohort by Haveman and colleagues [11] and the broader meta-analytic evidence [12,24], support the position that obesity should not be considered as a contraindication to robotic-assisted gynecologic surgery at experienced centers. Adequately powered multicenter studies are needed to confirm the safety equivalence suggested here and to better characterize the interaction between BMI, surgeon experience, and rare adverse events.

## Figures and Tables

**Table 1:** Patient demographics and preoperative characteristics.

Characteristic	Normal Weight (*n* = 51)	Overweight (*n* = 59)	Obese (*n* = 69)	*p*-Value
Age (years), Mean ± SD	49.2 ± 7.3	50.2 ± 8.5	49.1 ± 7.7	0.72
BMI (kg/m^2^), Mean ± SD	22.2 ± 1.6	26.8 ± 1.3	36.4 ± 6.6	<0.001
ASA 1/2/3, *n*	5/40/6	6/41/12	6/44/19	0.34
Prior surgeries ≥ 2, *n* (%)	10 (19.6%)	12 (20.3%)	10 (14.5%)	0.87
Smokers, *n* (%)	16 (31.4%)	15 (25.4%)	21 (30.4%)	0.75
Uterine weight (g) *	352.7 ± 354.4	432.5 ± 731.9	408.1 ± 425.6	0.618

* Uterine weight available for 153/179 patients.

**Table 2:** Perioperative outcomes by BMI group.

Outcome	Normal Weight (*n* = 51)	Overweight (*n* = 59)	Obese (*n* = 69)	*p*-Value
Operative time (min)	136.3 ± 68.7	146.7 ± 67.3	174.4 ± 74.3	0.009
EBL (mL)	600.8 ± 346.9	627.5 ± 573.4	824.3 ± 652.3	0.029
Postop Hb (g/dL)	11.4 ± 2.0	11.3 ± 1.9	11.4 ± 1.7	0.87
Postop CRP (mg/dL)	2.67 ± 3.13	3.11 ± 3.06	4.94 ± 4.99	0.004
Transfusion, *n* (%)	3 (5.9%)	3 (5.1%)	3 (4.3%)	0.93
Hospital stay (d), Median (IQR)	4.0 (3.0–5.0)	4.0 (2.5–5.0)	3.0 (2.0–4.0)	0.24

**Table 3:** Intraoperative and postoperative complications by BMI group.

Complication	Normal Weight (*n* = 51)	Overweight (*n* = 59)	Obese (*n* = 69)	*p*-Value
Intraoperative, *n* (%)	12 (23.5%)	9 (15.3%)	21 (30.4%)	0.13
Postoperative, *n* (%)	14 (27.5%)	17 (28.8%)	20 (29.0%)	0.91
Minor (CD I–II)	47 (92.2%)	58 (98.3%)	62 (89.9%)	0.15
Major (CD ≥ III)	4 (7.8%)	1 (1.7%)	7 (10.1%)	
Readmission ≤ 6 wk, *n* (%)	4 (7.8%)	2 (3.4%)	7 (10.1%)	0.33

## Data Availability

The data presented in this study are available on reasonable request from the corresponding author. The data are not publicly available due to privacy and ethical restrictions related to patient confidentiality.
